# Safety Profile and Radiographic and Clinical Outcomes of Stand-Alone 2-Level Anterior Lumbar Interbody Fusion: A Case Series of 41 Consecutive Patients

**DOI:** 10.7759/cureus.11684

**Published:** 2020-11-24

**Authors:** Osama Kashlan, Jason M Frerich, James G Malcolm, Matthew F Gary, Gerald E Rodts, Daniel Refai

**Affiliations:** 1 Neurosurgery, University of Michigan, Ann Arbor, USA; 2 Neurosurgery, Emory University School of Medicine, Atlanta, USA; 3 Orthopedic Surgery, Emory University School of Medicine, Atlanta, USA

**Keywords:** anterior lumbar interbody fusion, fusion, interbody fusion, lumbar spine, multilevel fusion

## Abstract

Objective: The use of stand-alone 2-level anterior lumbar interbody fusion (ALIF) for degenerative lumbar disease has been increasing as an alternative to routinely augmenting these constructs with posterior fixation or fusion. Despite the potential benefits of a stand-alone approach (decreased cost and operative time, decreased pain and early mobilization), there is a paucity of information regarding these operations in the literature. This investigation aimed to determine the safety profile, radiographic outcomes including fusion rates, improvement in preoperative pain, and spinopelvic parameter modification, for patients undergoing stand-alone 2-level ALIF.

Methods: This retrospective case series involved a chart review of all patients undergoing 2-level stand-alone ALIF at a single tertiary hospital from 2008 to 2018. Data included patient demographics, hospitalization, complications and radiological studies. Visual analog scale (VAS) back and leg scores were measured via patient-administered surveys preoperatively and up to 18 weeks postoperatively.

Results: Forty-one patients who underwent L4-S1 stand-alone ALIF were included. Sixteen (39%) of patients had undergone previous posterior lumbar surgery. Length of stay averaged 4.2 days. Complication rates were comparable to 1-level ALIF. Two patients required reoperation. Fusion rates were 100% for L4-5 and 94.4% for L5-S1. There was no significant change in lumbar lordosis (LL) or LL-pelvic incidence (PI), but there was improved segmental lordosis (SL) and disc height at L4-S1 on final follow-up imaging. There was also modest but statistically significant improvement in VAS back and leg scores.

Conclusions: Stand-alone 2-level ALIF is an option for a surgeon to perform in the absence of significant instability, even in the setting of prior posterior surgery. These procedures increase SL and disc height, but do not have the same effect on LL or LL-PI.

## Introduction

Lumbar interbody fusion can be performed from anterior (anterior lumbar interbody fusion [ALIF]), lateral (lateral or oblique lumbar interbody fusion [LLIF or OLIF]), or posterior (posterior or transforaminal lumbar interbody fusion [PLIF or TLIF]) approaches [1]. The anterior approach allows for a comprehensive ventral discectomy and straightforward endplate preparation, followed by direct graft insertion [2,3]. Comparable to posterior and lateral approaches, ALIF is associated with high fusion rates [1,4-6]. This approach allows substantial deformity correction and indirect neuroforaminal decompression, while also enabling early postoperative mobilization by sparing posterior spinal and psoas muscle dissection [7-11]. ALIF may be superior to TLIF, LLIF or OLIF at providing both segmental and overall lordosis correction [1,4,11,12]. The anterior approach has limitations however, as it is most suitable for the L4-5 and L5-S1 levels and provides only indirect neural element decompression. Complications can include visceral, ureteral, and vascular injuries, along with retrograde ejaculation secondary to sympathetic injury [13,14].

Stand-alone 2-level ALIF is an alternative to routinely augmenting multilevel anterior constructs with posterior fixation or fusion in patients without significant instability. However, the literature evaluating this technique is limited, especially in considering patients who have undergone prior posterior surgery. To our knowledge, no prospective or large-series retrospective studies are available. The potential benefits of stand-alone 2-level ALIF include early postoperative mobilization and high fusion rates, with satisfactory neuroforaminal height restoration and neurospinopelvic parameter improvement. Socioeconomic benefits may include decreased operating time, length of stay, and overall cost. We sought to evaluate stand-alone 2-level ALIF in a large comparatively homogenous series of patients, in the absence of significant instability, and even with a history of prior posterior lumbar surgery.

## Materials and methods

Institutional review board approval was obtained for this study; as a retrospective review of medical records and charts, the informed consent of patients was not sought. We performed a search of the operating room electronic scheduling record of a single tertiary hospital to locate all patients undergoing 2-level stand-alone ALIF from 2008 to 2018. A retrospective review of each patient’s chart was performed to obtain information on the following patient demographics: gender, smoking status, presence of osteoporosis, history of previous posterior surgery, age at the time of surgery, body mass index and pain characteristics. Details about the operation and hospitalization were collected, including the use of bone morphogenic protein-2 (BMP-2) (Medtronic, Dublin, Ireland) and length of stay. Polyetheretherketone (PEEK) (Globus, Audubon, PA, USA) and titanium-coated PEEK (Aesculap, Tuttlingen, Germany) implants were used. Immediate postoperative and long-term complications were also collected, including re-operation, retrograde ejaculation, intraoperative vessel injury, hardware complication, adjacent segment disease, urinary symptoms, deep vein thrombosis, and cerebrospinal fluid (CSF) leak.

All preoperative, immediate postoperative, and final standing radiographs, as well as computed tomography (CT) scans, for those patients included in the analysis were evaluated. A single spine surgeon measured all spinopelvic parameters to decrease the interobserver variability. Parameters measured were lumbar lordosis (LL, angle from the superior L1 endplate to the superior S1 endplate), segmental lordosis (SL, angle from the superior L4 endplate to the superior S1 endplate), sacral slope (SS), pelvic tilt (PT), pelvic incidence (PI), L4-5 anterior disc height, L4-5 posterior disc height, L5-S1 anterior disc height, and L5-S1 posterior disc height. X-rays were utilized to evaluate for fusion in the interbody cage as to limit unnecessary ionizing radiation. When appropriate, CT scans were obtained and evaluated for the presence of interbody fusion. Only patients with imaging studies at least 40 weeks (approximately nine months) from surgery were included in the fusion analysis. A paired t-test (SAS Institute, Cary, NC, USA) was utilized to determine differences between the means of spinopelvic parameters at the immediate postoperative period and on final X-rays in comparison to the preoperative radiographs.

Voluntary electronic surveys were given to patients preoperatively and at every clinic appointment with questions about pain levels. These included a 10-point visual analog scale (VAS) for a patient’s maximum back pain, average back pain, maximum leg pain, and average leg pain. A paired t-test was used to determine differences between these scores at every clinic visit in comparison to the preoperative scores.

## Results

Patient demographics

Of the 41 patients who underwent L4-S1 ALIF, 22 were female and 19 were male. The average age at surgery was 51.9 years (95% confidence interval (CI): 47.6-56.2). The average body mass index was 28.1 (95% CI: 26.8-29.3). When asked about radicular or lower back pain as a percentage of total symptomology, patients noted that an average of 62.4% of their total symptoms were due to back pain (95% CI: 52.3-72.4). Only one patient was a smoker, and one patient had a history of osteopenia. Sixteen patients (39%) had a history of previous posterior lumbar surgery. Ten of these patients underwent bilateral laminectomies with or without discectomy at L4-5, L5-S1 or both. Five patients underwent hemilaminectomy or laminotomy with or without discectomy at L4-5, L5-S1 or both. One patient underwent a posterior fusion from L4-S1 with removal of posterior instrumentation and subsequent pseudarthrosis prior to presentation to our clinic. The demographic data are listed in Table 1.

**Table 1:** Preoperative baseline characteristics of patients undergoing 2-level stand-alone anterior lumbar interbody fusion

Demographic	No. Patients (%)
Gender	
Female	22 (53.7%)
Male	19 (46.3%)
Smoking status	
No	40 (97.6%)
Yes	1 (2.4%)
Osteopenia	
No	40 (97.6%)
Yes	1 (2.4%)
Previous posterior surgery	
No	25 (61.0%)
Yes	16 (39.0%)

Immediate postoperative and long-term details and complications

All approaches were performed through a transperitoneal approach with the aid of a vascular approach surgeon. The transperitoneal approach, even though not standard, was the choice of the approach surgeon. BMP-2 (8.2 mg per level) was used as an interbody graft in both cages and in all patients. The average length of stay was 4.2 days (95% CI: 3.6-4.8). Immediate postoperative and long-term complications are listed in Table 2.

**Table 2:** Perioperative and long-term complications for patients undergoing 2-level stand-alone anterior lumbar interbody fusion

Complication	No. Patients (%)
Re-operation	2/41 (4.88%)
Adjacent segment disease	3/41 (7.32%)
Ileus requiring nasogastric tube placement and/or prolonged hospital stay (>7 days)	3/41 (7.32%)
Permanent retrograde ejaculation	2/19 (10.5%)
Transient retrograde ejaculation	1/19 (5.26%)
Transient urinary hesitancy and erectile dysfunction	1/41 (2.44%)
Transient urinary retention	2/41 (4.88%)
Intraoperative vessel Injury	3/41 (7.32%)
Hardware complication	2/41 (4.88%)
Deep vein thrombosis	2/41 (4.88%)
Cerebrospinal fluid leak	1/41 (2.44%)

Overall, 17 of 41 of patients (41.5%) had at least a minor complication after surgery, which included re-operation, adjacent segment disease, prolonged ileus, transient or permanent retrograde ejaculation, transient urinary retention/hesitancy, hardware complication, deep vein thrombosis (DVT), intraoperative vessel injury, and cerebrospinal fluid leak. Three patients (7.32%) developed adjacent segment degeneration, with two treated conservatively and another treated with surgery. Two patients (4.88%) underwent re-operation: one for early hardware failure at L5-S1 with subsequent S1 endplate fracture, and another for early adjacent segment degeneration at L3-4. Three patients (7.32%) developed a clinically significant ileus requiring nasogastric tube placement or a prolonged hospital stay greater than seven days. Two males (10.5%) had permanent retrograde ejaculation, while another (5.26%) had transient retrograde ejaculation. Three patients (7.32%) had transient urinary dysfunction, sexual dysfunction or both. Three patients (7.32%) had an intraoperative vessel injury: two caused by retractors injuring the vena cava or iliac vein and one iliac vein injury that occurred during screw placement. There were two (4.88%) hardware complications: lateral breach of an L4 screw discovered and removed intraoperatively with a resultant radiculopathy treated medically, and an L4-5 interbody cage placement causing L4 body fracture that was managed conservatively. Two patients (4.88%) developed DVT. One patient (2.44%), who had a history of prior L4-5 discectomy, sustained an intraoperative CSF leak during discectomy at the L4-5 level.

Fusion rates and spinopelvic parameters

Thirty-six of 41 patients (87.8%) had X-rays, CT scans or both performed at least 40 weeks postoperatively. Average follow-up was 59.9 weeks (95% CI: 48.5-71.3). One patient was lost to follow-up immediately after surgery. Two patients were lost to follow-up after their six-week postoperative visit. Two patients were removed due to re-operations with subsequent posterior instrumentation and fusion. All 36 eligible patients fused at L4-5. Thirty-four of the 36 patients (94.4%) fused at L5-S1. In the two patients with L5-S1 pseudarthrosis, there was fusion mass formation at L4-5 but not at L5-S1. This was also indicated on their X-rays. X-rays alone were sufficient to confirm fusion for 20 patients. CT scans confirmed fusion in 14 of 16 patients.

Spinopelvic parameters were determined based on imaging acquired at preoperative, immediate postoperative and final visit intervals; they are listed in Table 3. Thirty-four patients had preoperative standing X-rays. Seven patients either did not have standing X-rays or had only flexion-extension X-rays. Thirty-eight patients had final X-rays. Three patients were removed from final X-ray analysis: two secondary to early reoperation and one who had no follow-up after discharge from the hospital. Of the final X-rays of 38 patients, two were obtained at the six-week visit, 16 at the 40-week visit and 20 at a visit greater than 52 weeks. There was a significant decrease of 5.0 degrees in LL between preoperative and immediate postoperative X-rays, and a significant decrease in SS of 2.1 degrees in the immediate postoperative period. However, there was no difference between preoperative and final LL or SS. There was a significant increase in SL of 7.7 degrees in the immediate postoperative period. This difference decreased to 3.9 degrees on final X-rays, though still statistically significant. There was no significant difference in PT between the three time periods. Finally, there was a significant increase in anterior and posterior disc heights at both L4-5 and L5-S1 immediately postoperatively. This increase continued to be statistically significant, though diminished, on the final radiographs. When evaluating PI-LL balance (PI and LL being within 10 degrees of one another), 52.6% of patients were balanced preoperatively, and 68.4% were balanced at final follow-up. This trend was not statistically significant. The average PI-LL difference was 10.5 degrees preoperatively and 8.6 degrees postoperatively, which was also not statistically significant.

**Table 3:** Spinopelvic parameters for patients undergoing 2-level stand-alone anterior lumbar interbody fusion (ALIF) as measured in preoperative, immediate postoperative and final standing radiographs Asterisk (*) denotes statistical significance with p < 0.05.

Parameter		Preoperative Mean ± SD		Postoperative Mean ± SD		Final Mean ± SD
Lumbar lordosis (°)		48.2 ± 8.42 (n = 34)		43.2* ± 10.8 (n = 41)		50.8 ± 8.44 (n = 38)
Segmental lordosis (°)		31.5 ± 9.41 (n = 34)		39.2* ± 8.00 (n = 41)		35.4* ± 6.51 (n = 38)
Sacral slope (°)		35.5 ± 7.26 (n = 34)		33.4* ± 8.06 (n = 41)		36.4 ± 6.63 (n = 38)
Pelvic tilt (°)		17.9 ± 9.35 (n = 23)		21.4 ± 9.31 (n = 21)		17.2 ± 6.60 (n = 33)
L4-5 Anterior disk height (mm)		11.5 ± 4.59 (n = 34)		17.2* ± 3.82 (n = 41)		15.9* ± 3.89 (n = 38)
L4-5 Posterior disk height (mm)		5.47 ± 2.27 (n = 34)		8.19* ± 2.55 (n = 41)		7.27* ± 2.30 (n = 38)
L5-S1 Anterior disk height (mm)		11.1 ± 4.34 (n = 34)		19.0* ± 2.31 (n = 41)		17.9* ± 2.87 (n = 38)
L5-S1 Posterior disk height (mm)		3.77 ± 1.59 (n = 34)		6.12* ± 1.91 (n = 41)		6.02* ± 1.69 (n = 38)

Clinical pain outcomes

Only 32 patients responded to VAS back pain questions preoperatively, and only 27 and 26 patients responded to the VAS maximum leg pain and average leg pain questions, respectively. At the six-week postoperative visit, the number of respondents decreased to 25 and 18 for VAS back (both maximum and average) and VAS leg pain, respectively. At 18 weeks, 23 patients responded to the VAS back pain (both measures). At the same time point, 19 patients responded to the VAS maximum leg pain and 16 patients responded to the VAS average leg pain. The response rate dropped much lower at the 11-month visit and therefore those results are not presented.

**Figure 1:**
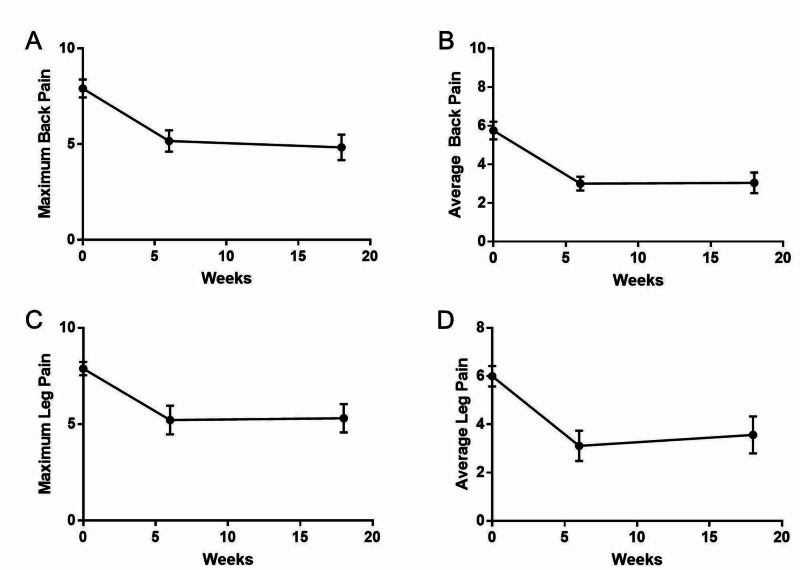
Graphical depiction of visual analog scale (VAS) scores for back and leg pain. (A) Maximum back pain, (B) average back pain, (C) maximum leg pain, and (D) average leg pain all statistically improved postoperatively at the 6-week and 18-week visits (p < 0.05). Error bars show standard error of the mean.

The VAS maximum back pain score preoperatively was 7.9 (standard error of the mean = 0.47), decreased to 5.2 (0.56) at six weeks, and further to 4.8 (0.67) at 18 weeks. The VAS average back pain score preoperatively was 5.8 (0.46), decreased to 3.0 (0.36) at six weeks, and remained stable at 3.0 (0.53) at 18 weeks. The VAS maximum leg pain score preoperatively was 7.9 (0.34), decreased to 5.2 (0.74) at six weeks postoperatively, and then increased slightly to 5.3 (0.74) at 18 weeks. The VAS average leg pain score preoperatively was 6.0 (0.43), decreased to 3.1 (0.626) at six weeks postoperatively, and then increased slightly to 3.6 (0.77) at 18 weeks. All values from the four measures at the two postoperative time points were statistically improved (p < 0.05) when compared to the preoperative measures. This trend is graphically shown in Figure 1.

## Discussion

We present our experience with performing 2-level stand-alone ALIF in patients with symptomatic degenerative lumbar disease without significant instability. Avoiding posterior fixation or fusion in these patients decreases overall cost and operative time, while enabling early postoperative mobilization and reducing length of stay [15]. We found an average length of stay of 4.2 days, well in accordance with the national average of 5.1 days for ALIF and considerably below the average of six days for combined anterior-posterior fusion approaches [15]. However, though this technique is utilized on a widespread basis in spine surgery, most of the evidence regarding this procedure comes from heterogeneous studies or meta-analyses that combine patients undergoing 1-level and multilevel stand-alone ALIF. As such, our study describes the largest series of 2-level stand-alone ALIF in the literature. We demonstrate that this intervention achieves high fusion rates without the need for posterior instrumentation, even in those patients who had undergone previous posterior lumbar surgery, provided there is no significant instability. Based on our experience, significant instability likely is any mobile spondylolisthesis with greater than 3 mm of translation between flexion-extension X-rays. However, this claim would need significant testing with prospective, larger studies for validation. Patients with significant instability or concerns regarding the strength of anterior fixation would likely require a posterior fixation or fusion procedure and would not be candidates for a stand-alone 2-level ALIF.

**Figure 2:**
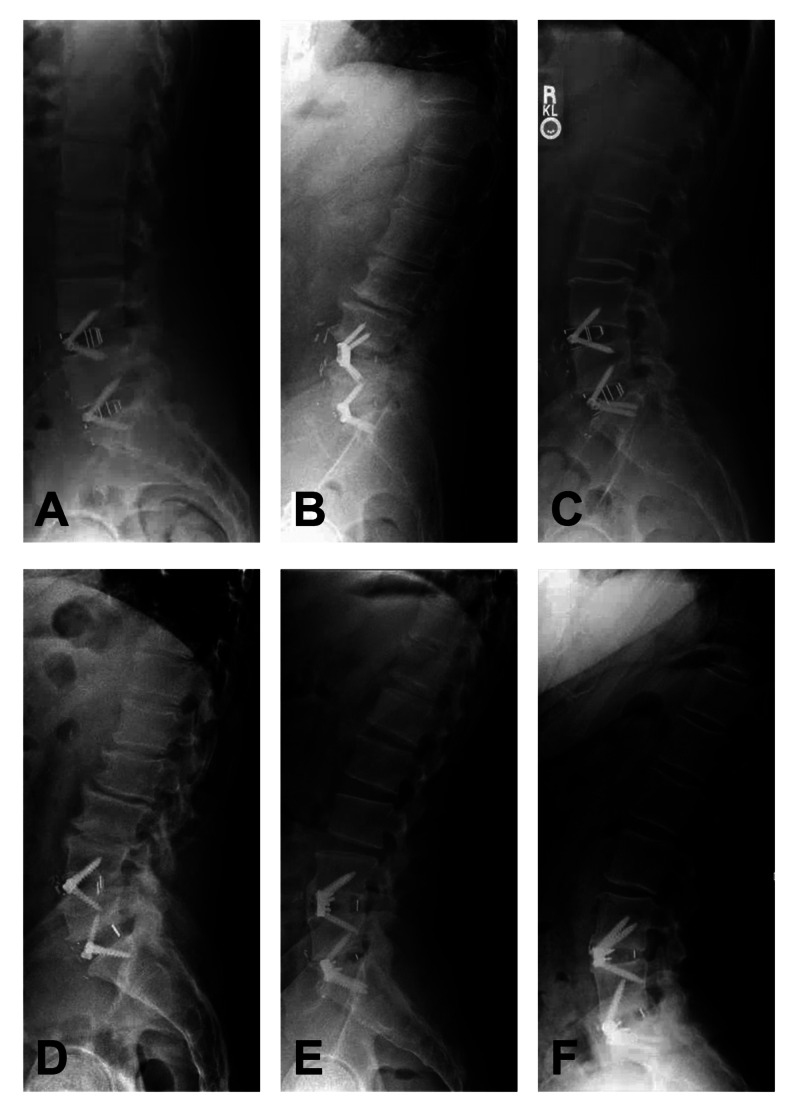
Postoperative (>6 months) upright radiographs of patients with a history of prior posterior lumbar surgery and after undergoing stand-alone L4-S1 anterior lumbar interbody fusion (ALIF). Patients had a history of either single-level or multilevel lumbosacral laminectomy: (A) L4-S1, (B) L3-S1, (C) L4-5, (D) L3-5, (E) L4-5 and (F) L5. These examples illustrate the safety in performing a stand-alone L4-S1 ALIF in the setting of prior posterior surgery.

A high fusion rate was achieved even with 39% of patients having undergone previous posterior surgeries, most of which were complete laminectomies rather than hemilaminectomies or laminotomies (Figure 2). This result suggests that disruption to the posterior tension band is not an absolute contraindication to performing 2-level stand-alone ALIF. Moreover, four patients had a preoperative grade-I spondylolisthesis at L4-5, three of which worsened just slightly on flexion-extension X-rays. The fourth patient did not have dynamic X-ray imaging available, though comparing upright to supine imaging modalities did not demonstrate a change in the amount of anterolisthesis. One of these four patients was lost to follow-up, but the other three fused at the L4-5 level (one of whom had an L4-5 unilateral iatrogenic pars fracture from a previous laminectomy). None of our patients had spondylolysis at either the L4-5 or L5-S1 levels. However, one patient in the cohort preoperatively had a mobile grade-I spondylolisthesis at L5-S1 that worsened to an almost grade-II spondylolisthesis upon standing. Postoperatively, this patient developed an S1 endplate fracture with anterior migration of the L5-S1 cage, ultimately requiring posterior instrumented fusion two months later. In combining the findings above, 2-level stand-alone ALIF achieves a high fusion rate in patients with a history of previous posterior surgery, provided the patient does not have gross preoperative instability, especially at L5-S1.

The high fusion rates observed in this study are comparable with those of the interbody fusion literature. However, they might be inflated by patient selection (only one smoker and one patient with osteopenia) and using BMP-2 in all cages. Our dose of BMP-2 per level is within the range described in the literature [16]. Nonetheless, two patients developed pseudarthrosis at L5-S1, likely due to increased forces at the lumbosacral junction compared to L4-5. In one of these patients, who developed pseudarthrosis at L5-S1 nine months postoperatively, we had initially planned to perform posterior percutaneous fixation in a delayed fashion due to a significant scoliotic deformity at L4-S1. However, the patient opted against continuing with the posterior surgery due to improvement in symptoms after the anterior procedure. The patient was subsequently lost to follow-up. The second patient developed pseudarthrosis diagnosed at 15 months postoperatively. Even though she had significant symptoms, she opted for continued conservative management and surveillance imaging with the hope of achieving delayed fusion. Of note, this patient had a high PI of 74.9 degrees. In addition, another patient with a high PI of 68.0 degrees reached delayed L5-S1 fusion at 18 months. These findings suggest that 2-level stand-alone ALIF should probably be supplemented with posterior percutaneous fixation or fusion in patients with a high PI. However, this is not an absolute recommendation as there was also a patient in our cohort with a PI of 75.2 degrees who had fused by one year. Further prospective studies are needed to determine if a PI threshold exists that makes posterior supplementation necessary.

We found a risk of adjacent segment degeneration after 2-level stand-alone ALIF comparable to that described in the literature. Three patients (7.32%) developed adjacent segment degeneration, two in a delayed fashion (at two years and 15 months). Both patients had developed adequate fusion at L4-S1 and ultimately opted for conservative management. The third patient developed significant adjacent segment degeneration two months postoperatively and required an L3-4 TLIF with L3-S1 posterior instrumented fusion. However, this outcome was not entirely unexpected as preoperative imaging demonstrated significant disc disease at L3-4 with an associated disc herniation causing mild stenosis. Because of this patient’s young age of 25 years, we opted to initially manage only the most symptomatic segments and performed an L4-S1 ALIF. We perhaps should have initially been more aggressive with treatment of the L3-4 disc space.

The most problematic perioperative complications associated with the anterior spinal approach are ileus, vascular injury, bowel injury, DVT due to venous retraction, and damage to the sympathetic plexus causing retrograde ejaculation in men. Our low rate of ileus, even with utilizing our vascular approach surgeon’s preferred transperitoneal approach, is likely due to prophylactic placement of the patients on scheduled intravenous prokinetic drugs, meticulous electrolyte replacement and strict diet advancement postoperatively. There were no bowel injuries in the cohort, likely owing to the experience of the approach surgeon. We did have three patients (7.32%) with an intraoperative vessel injury, and the vascular surgeon repaired these injuries intraoperatively with no significant blood loss or patient morbidity. This finding demonstrates the importance of having an experienced approach surgeon present and involved in the operation from beginning to end. Two patients developed DVT postoperatively, one of whom had a remote history of DVT with associated pulmonary emboli and was bridged off his anticoagulation for our surgery. The other was unprovoked. Neither of these patients developed pulmonary embolus and both were successfully treated with anticoagulation. To help prevent against DVT, we administer 5000 units of heparin subcutaneously preoperatively; we also minimize venous retraction time and have retraction “breaks” every few minutes to prevent venous stasis. Lastly, our permanent retrograde ejaculation rate of 10.5% is comparable to that seen in the literature for 1- or 2-level ALIF with concurrent use of BMP-2 [17, 18]. This result suggests that performing a 2-level ALIF does not specifically place patients at an increased risk of developing this complication when compared to a 1-level ALIF. It is important to note that a meta-analysis of the two techniques showed a higher postoperative retrograde ejaculation rate and a trend towards higher overall complication rates in the transperitoneal approach (which is what we used) versus the retroperitoneal approach [19]. The approach surgeon at our institution is an experienced vascular surgeon, which might explain our complication rates being similar to the complication rates following a retroperitoneal approach in the literature. Lastly, a meta-analysis of BMP-2 in ALIF demonstrated a weak trend between likelihood of complications (retrograde ejaculation, endplate resorption, and graft subsidence) and dosing of BMP-2. Therefore, decreasing the dose of BMP-2 further might decrease complication rates further [16].

There is some controversy regarding the effect of ALIF on spinopelvic parameters. We found that 2-level ALIF did not significantly alter most parameters on final X-rays, though we did find an increase in SL of 3.9 degrees, as demonstrated in previously reported studies [7]. The most notable radiographic change following 2-level ALIF was increased anterior and posterior disc height at both L4-5 and L5-S1. Subsidence expectedly occurred at operated levels between the immediate postoperative and final X-rays, with a slight decrease in both final SL and disc height measurements. However, performing 2-level ALIF did not ultimately alter final LL, SS, PT or LL-PI parameters in a significant way. We suspect the transient decreases in LL and SS, observed immediately postoperatively, were due to patient discomfort and a subsequently associated positional distortion. Nonetheless, the spinopelvic parameter findings suggest that 2-level stand-alone ALIF should not be utilized as an isolated deformity operation, and this intervention requires supplementation with posterior osteotomies and compression to achieve significant corrections to LL. This is supported by the fact that, although trending toward ideal (≤10 degrees), we did not have a statistically significant change in LL-PI or the number of patients with corrected LL-PI mismatch after surgery [20]. Further large prospective studies are needed to assess this assertion and to determine what spinopelvic parameter changes may be tied to clinical outcomes. There is increasing evidence to suggest that patients with ideal LL-PI values postoperatively have reduced back pain [20-22].

With regards to clinical outcomes after a 2-level stand-alone ALIF, our study showed statistically significant improvement in back and leg pain postoperatively. Even though statistically significant, the clinical effect in our study was modest, as the patients continued to have back and leg pain-albeit at a lower level. However, this result should be seen with the caveat of a low patient response rate, which is almost certainly due to the surveys being completely voluntary and some patients likely decided not to take the time to fill them out. This significant limitation of our study likely biases the results, especially if there is a higher propensity of patients with good outcomes to disregard the survey when compared to patients with poor outcomes, or vice versa.

This study is the largest to date assessing radiographic outcomes associated with 2-level stand-alone ALIF, though the findings remain limited by the retrospective nature of the analysis and by the number of patients. The paucity of functional outcomes information is the most glaring limitation of this study and will be addressed in subsequent studies. Moreover, though our study had an acceptable follow-up rate (87.8%) with 36 of 41 eligible patients reaching an average of 59.9 weeks, the loss of some patients and lack of long-term data may decrease our stated rates of adjacent segment degeneration and reoperation. As such, we listed absolute numbers for each characteristic studied in addition to percentages. Another limitation is the fact that most of our fusion analysis comes from evaluating X-rays, which is not ideal. Nonetheless, our practice evolved to this pattern to minimize radiation in patients who are doing well clinically. Lastly, the goal of this project was not to discuss or help determine the indications for when to perform an ALIF versus TLIF. The goal was to show that it is reasonable and possible in a select patient subgroup to perform a stand-alone 2-level ALIF rather than a 2-level ALIF with posterior fixation or fusion. On the same note, in our practice, the patient population requiring ALIF in addition to posterior fixation differs from the population of patients who are candidates for a 2-level stand-alone ALIF. This is the gist of this manuscript. For this reason, we decided to present these data as a case series and not as a comparative study to TLIF patients or ALIF patient with posterior fixation or fusion. 

## Conclusions

Utilizing a retrospective analysis, we demonstrate that performing 2-level stand-alone ALIF in patients without significant instability is a valid option and achieves high fusion rates even in the setting of previous posterior lumbar surgery. The findings also illustrate the lack of a significant change in LL, SS and PT. However, 2-level ALIF does significantly increase SL and disc height at operated levels. These changes are greatest immediately after surgery but decrease in magnitude due to subsidence on final radiographs. Further prospective studies, including higher patient numbers and functional outcomes, are required to substantiate our findings.
